# Imaging Findings in Neonates With Congenital Pyriform Sinus Fistula: A Retrospective Study of 45 Cases

**DOI:** 10.3389/fped.2021.721128

**Published:** 2021-11-02

**Authors:** Li Li, Dong-Ji-Hui Zhao, Tao-Yue Yao, Yong-Hua Xiang, Hong Liu, Qiu-Hong Ma, Ke Jin, Si-Ping He

**Affiliations:** ^1^Department of Radiology, Hunan Children's Hospital, University of South China, Changsha, China; ^2^Otorhinolaryngology, Head and Neck Surgery, Hunan Children's Hospital, University of South China, Changsha, China; ^3^Department of Ultrasound, Hunan Children's Hospital, University of South China, Changsha, China

**Keywords:** neonates, diagnosis, computed tomography, ultrasonography, Congenital pyriform sinus fistula

## Abstract

**Background:** Congenital pyriform sinus fistula (CPSF) is a rare branchial cleft deformity. The characteristics and management of CPSF in neonates are different from those in children or adults, and a comprehensive understanding of the imaging features of neonatal CPSF can facilitate its preoperative diagnosis. Thus, the aim of this study was to summarize the ultrasonography (US) and CT imaging findings of CPSF in neonates.

**Methods:** Forty-five full-term neonates with CPSF, confirmed by pathology after surgical resection from January 2012 to October 2020, were included in this retrospective study. All patients underwent preoperative cervical US and contrast-enhanced CT examinations, and the imaging findings were analyzed.

**Results:** Forty-six cervical cystic masses were found in 45 neonates, including one case with bilateral lesions, three cases with lesions on the right side, and 41 cases on the left side. Both US and CT detected neck abnormality among all cases, while the diagnostic accuracy of US (15/46, 32.6%) was lower than that of CT (42/46, 91.3%). Moreover, CT showed significantly higher detection rates of intralesional air bubbles, involvement of the ipsilateral thyroid, deviation of the airway, and expansion into the mediastinal and retropharyngeal space compared with the US. As the age increased, it was more likely to present some features including the absence of air-containing, thick cyst wall, and poorly defined border (ρ <0.05).

**Conclusion:** CPSF in the neonates showed distinctive imaging findings on contrast-enhanced CT scan, which provides important supplementary information for the diagnosis of CPSF after the initial US examination.

## Introduction

Congenital pyriform sinus fistula (CPSF) is a rare branchial cleft deformity, which originates from the incomplete occlusion of the third and fourth branchial cleft, accounting for 2–10% of branchial cleft deformities (1, 2). However, with the development of imaging examinations and increasing knowledge about it, the incidence is on the rise (3). During the embryonic period, the dorsal side of the third and fourth branchial cleft develop into inferior parathyroid and superior parathyroid glands, and the ventral side develops into the thymus and thyroid (4). CPSF is formed in the occasions where the branchial cleft is not completely degenerated. CPSF becomes a collective term for both malformations due to the same clinical manifestation and management for the third and fourth branchial cleft deformities. About 80% of cases have their onset in infancy or childhood (5, 6).

In general, confirmation of an internal orifice in the pyriform fossa through suspension laryngoscope is the diagnostic gold standard for CPSF (7). However, the suspension laryngoscope procedure can only be implemented under general anesthesia and usually is performed as a step in open radical surgery. The preliminary diagnosis of CSPF by preoperative imaging examinations is important. Insufficient understanding of the clinical and imaging manifestations may easily lead to misdiagnosis and delay the subsequent treatment (8–10). The clinical symptoms and imaging findings of CPSF in neonates differ from those in children or adults (11). However, there are quite a few empirical studies or large-scale reports on CPSF in neonates (9, 12). The present study aimed to summarize the ultrasonography (US) and CT findings of 45 neonates with CPSF and to investigate the radiological characteristics of CPSF for early diagnosis and treatment.

## Materials and Methods

### Study Design and Patients

**Figure 1:**
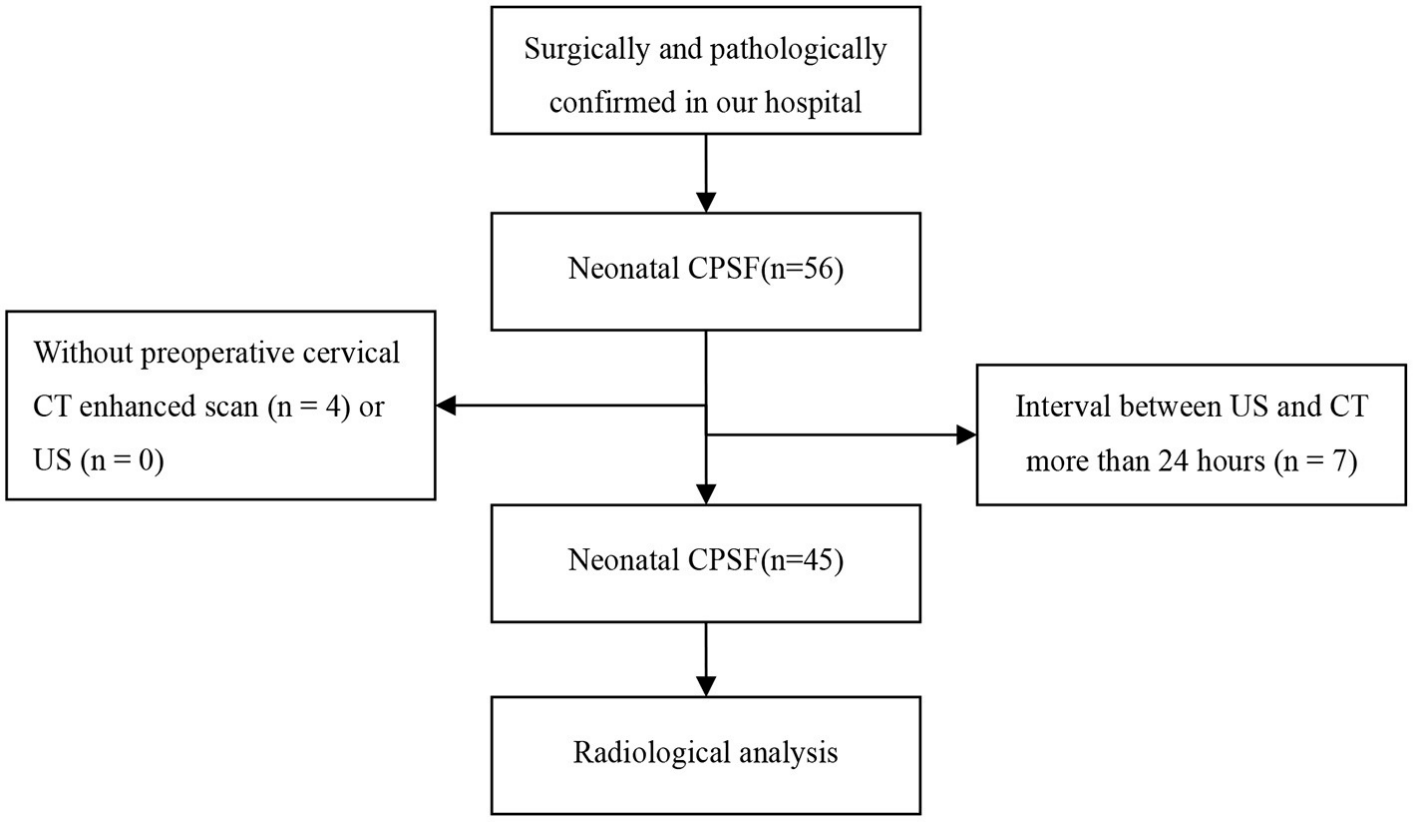
Flowchart of the present study.

This study was approved by our institutional review board and patient consent was waived. The data of patients confirmed with CPSF in our hospital from January 2012 to October 2020 were retrospectively reviewed. A flowchart of the patients enrolled for analysis, along with numbers of excluded patients and the reasons for their exclusion, is shown in Figure 1. The confirmation of the internal fistula orifice served as the standard for diagnosis of CPSF (7). In all cases, the diagnosis of CPSF was made by an open surgery on the basis of the existence of the internal fistula orifice and further confirmed pathologically. For each patient, medical records, including demographics, clinical manifestations, and CT and US findings, were reviewed.

### Imaging Examination

Patients were examined for neck mass in the supine position using a Philips Q7 color Doppler US diagnostic instrument with the L12-3 linear array probe (the frequency range of which is 3–12 MHz).

The Dutch Philips 64-row Brilliance double helix CT scanner was used for scanning. Fasting (no food or liquid) was required 1 h before scanning. Newborns were made to cry loudly for 10–20 s while being orally sedated with 10% chloral hydrate (0.5 ml/kg) at 30 min before the scan.

Plain scans and enhanced scans were carried out by CT. Routine CT scanning ranged from the skull base to the thoracic entrance level (the enhanced scan should reach the level of the fourth thoracic vertebra to ensure that the lesion is fully displayed if the lesion was beyond the scope of the plain scan). Iohexol (iodine-containing 320 mg/ml), a nonionic contrast agent, was injected through the scalp vein with an indwelling needle through a high-pressure syringe. The injection flow rate was 1.0 ml/kg and the injection dose was 10 ml. The scanning parameters were as follows: tube voltage, 100 kV; tube current, 80–100 mA; layer thickness, 3 mm; layer spacing, 3 mm; pitch, 1.5 sagittal and coronal images were reconstructed. We provided radiation protection to the newborn to avoid unnecessary damage to non-examination areas.

### Imaging Evaluation

The US/CT images were independently reviewed by two experienced pediatric sonographers/radiologists respectively. Differences in assessments by the two sonographers/radiologists were resolved by consensus, including the opinion of a third board-certified pediatric radiologist (more than 20 years of clinical experience in sonographers/pediatric radiology). The key imaging features of a cervical lesion were analyzed, including the location, size (transverse diameter × anteroposterior diameter × vertical diameter), morphology, internal echogenicity, blood flow signal, density, the enhancement pattern, and the relationship with the adjacent tissue structure of the lesion. Enhancement of the wall was regarded as low-, iso-, or high-attenuation compared with the adjacent muscle in enhancement scan. Given the differences in the characteristics and management of CPSF in patients of different ages (9, 13), we hypothesized that the imaging features of CPSF may differ in neonates of different ages. According to the age factor (1–7 days, 8–14 days, 15–28 days), therefore, we analyzed the CT images in neonates of different ages for the presence or absence of air-containing in cyst, whether the cyst wall was thick (>1 mm) or thin ( ≤ 1 mm), and whether the border of the lesion was well or poorly defined.

### Statistical Analysis

Statistical analyses were performed using the SPSS software version 22 (SPSS Inc., Chicago, IL, USA). The measurement data were expressed by mean ± SD ($\bar{x}$ ± s), and the counting data were expressed by frequency and percentage [*n* (%)]. The difference of rate was compared by trend χ^2^ test, and the test level was α = 0.05. A value of *p* < 0.05 was considered statistically significant.

## Results

### Clinical Manifestation

According to the study design, 45 neonate patients (23 males and 22 females) with surgically and pathologically proven CPSF were enrolled in this study, as shown in Figure 1. The age of the patients ranged from 1 to 28 days at the time of preoperative US and CT, with a median age of 11 days and an average age of 13.2 ± 7.5 days. All 45 cases were full-term newborns and came to the hospital for medical treatment due to finding masses in the anterior cervical region, with normal skin color, smooth surface, soft surface, and no tenderness. One case was found by the prenatal US at 35 weeks of gestation. Ten cases presented with shortness of breath, 31 with pulmonary infection, and 35 with progressive enlargement of the mass. The levels of C-reactive protein (CRP) in all neonates increased by different degrees (25 ±11 mg/L, normal range ≤ 6 mg/L).

### Radiological Findings

**Figure 2:**
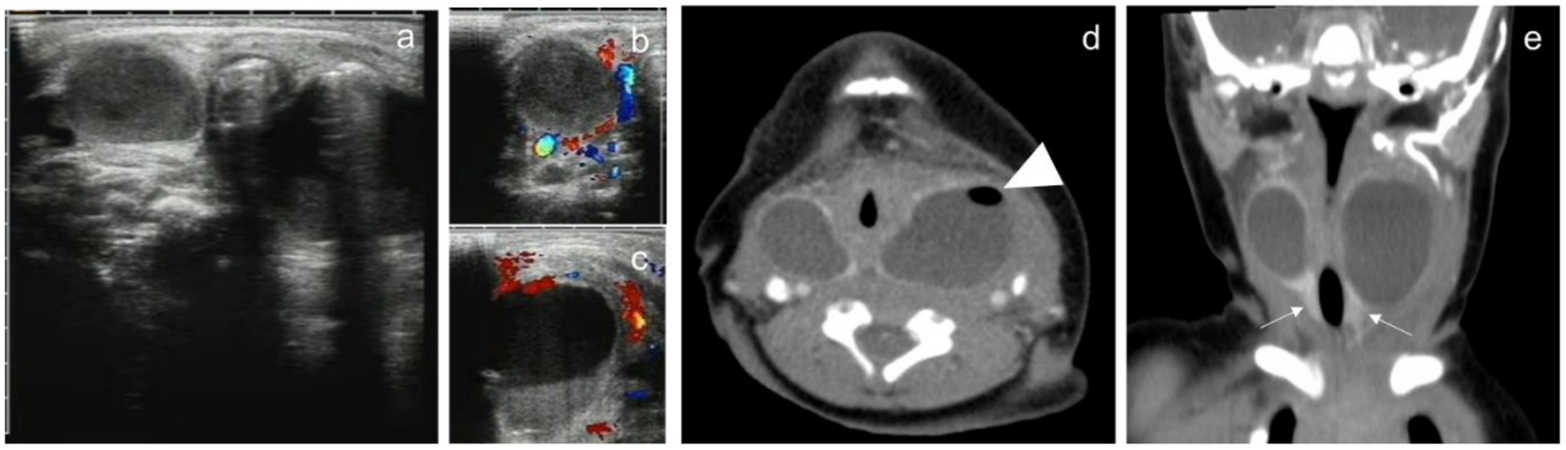
A 3-day male newborn with bilateral neck CPSF. Both US and contrast-enhanced CT showed bilateral cervical cystic masses with no septum. US presented inhomogeneous cystic hypoechoic with air-bubble containing **(a)**. CDFI: there was no blood flow in the lesions **(b,c)**. Contrast-enhanced CT showed the mass in the bilateral cervical visceral space penetrated into the retropharyngeal space, the wall of the cyst was thick and high attenuation, the border was well defined, and small bubbles could be seen in the cystic mass of the left neck (**d**, arrowhead). Coronal images showed bilateral cervical lesions involved the thyroid gland, and the thyroid enhancement density was homogeneous (**e**, arrow).

**Figure 3:**
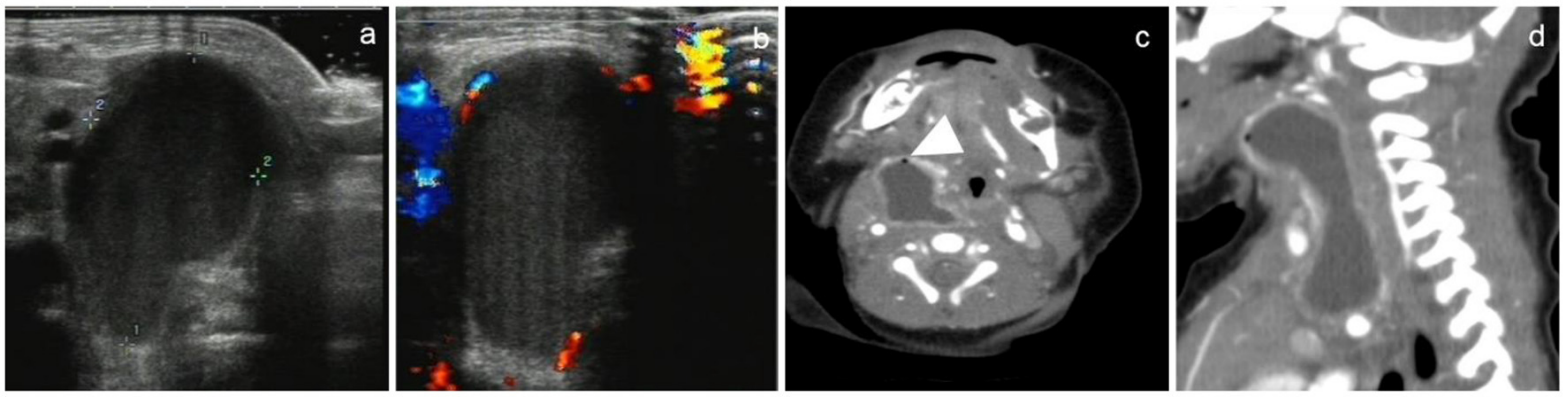
A 15-day-old female newborn with CPSF on the right neck. US and CT showed a non-septal cystic mass in the right neck. US showed an inhomogeneous mass with low echogenicity, in which there were no air bubbles **(a)**. Color Doppler showed there was no blood flow sonogram in the cyst **(b)**. CT showed that the cyst wall was thick and high attenuation, the border of the cyst was poorly defined, and tiny air bubbles could be seen in the cyst (arrowhead). The cystic mass extended laterally into the retropharyngeal space **(c)** and longitudinally into the anterior mediastinum **(d)**.

**Figure 4:**
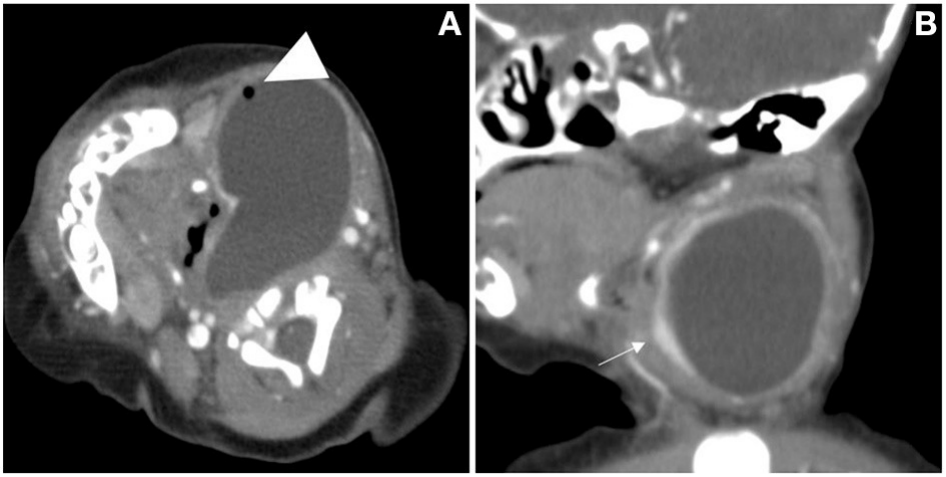
The cervical CT images of a 7-day-old girl. A cystic mass without septum was demonstrated in the left neck, small air bubbles can be seen inside (arrowhead), the wall of the cyst was thin and iso attenuation, and the border was well defined. The cystic mass, shaping as a “comma,” extended laterally into the retropharyngeal space and the airway was deviated **(A)**. The left thyroid gland lobe was involved and pushed anteriorly on sagittal view (**B**, arrow).

There were 46 cystic masses with no septum in all cases, which were detected by both US and CT. One case was found with bilateral neck cystic mass (Figure 2), 3 cases were found with right neck cystic masses (Figure 3), and the other 41 cases were found with left neck cystic masses (Figure 4). Based on the findings of the US (Table 1), only 15 cystic masses with air bubbles were diagnosed as CPSF (15/46), while none of the others could be definitively diagnosed. Typical ultrasonographic findings were cystic masses with inhomogeneous low echogenicity and poor penetration of internal echogenicity in the neck with or without air in the lesions. It was also shown that the cystic mass compressed against the adjacent airway, carotid artery, and sternocleidomastoid muscle. Moreover, a small portion of the cystic mass could develop into the retropharyngeal space. Lastly, the border of the cyst was well or poorly defined. Color Doppler flow imaging (CDFI) showed that there was no blood flow in the lesions.

**Table 1:** US findings of 46 lesions of CSPF.

**US findings**		**Value**
Neck position	Right/left	4/42
Echogenicity	Inhomogeneous cystic hypoechoic	46
Cyst wall	Thin/thick	20/26
Border	Well/poorly defined	21/25
Size (cm)	Transverse/anteroposterior/ vertical diameter (median)	3.3/3.0/3.8
Air bubbles in cysts	Present/absent	15/31
Involvement of thyroid gland	Present/absent	30/16
Airway compression/displacement	Present/absent	28/18
Into retropharyngeal space	Present/absent	18/28
Into mediastinum	Present/absent	4/42

**Figure 5:**
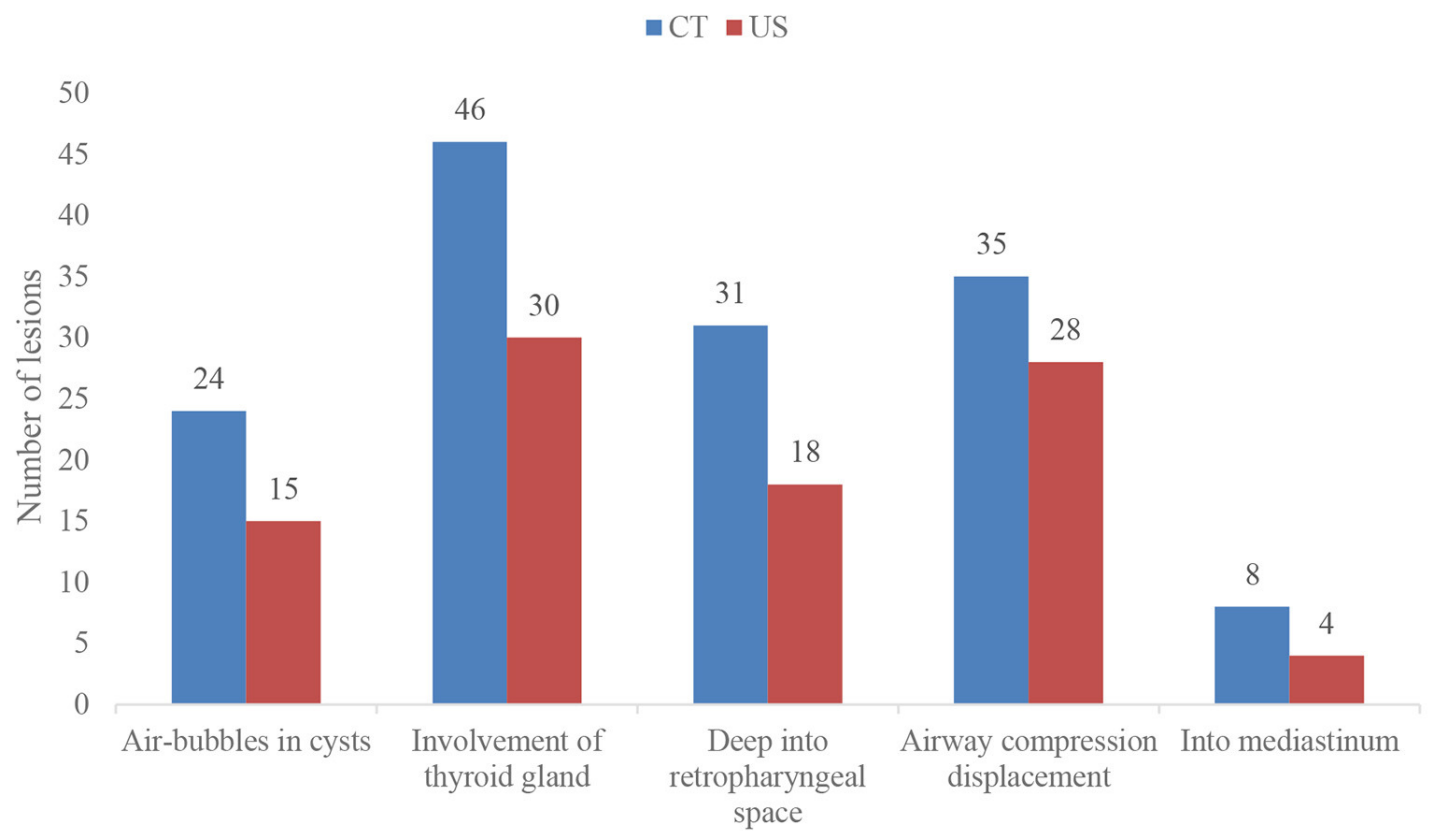
Comparison of CT and US imaging findings of CPSF.

Based on the findings of CT (Table 2), except for 4 lesions located in the right neck, 42 lesions were diagnosed as CPSF (42/46). Forty-six lesions located in the visceral space, resulting in the deviation of the trachea, the carotid space, and the sternocleidomastoid muscle. The vertical diameter was the largest diameter of the lesions. Most of the cysts were shaped into “commas,” which shifted deeper into the retropharyngeal space. The cyst wall showed iso-high attenuation in contrast-enhanced scan. The detection rates of air in the cyst, involvement with the ipsilateral thyroid, and extension into the mediastinum and retropharyngeal space were higher than those of the US (Figure 5). There were significant differences in the presence of air-containing, the cyst wall, and the border of the cyst exhibited in patients of different ages (Table 3). More specifically, older age was associated with the absence of air bubbles and thick cyst wall, as well as a poorly defined border.

**Table 2:** CT findings of 46 lesions of CSPF.

**CT findings**		**Value**
Neck position	Right/left	4/42
Density	Cystic fluid density	46
Cyst wall	Thin/thick	20/26
Border	Well/poorly defined	21/25
Size (cm)	Transverse/anteroposterior/ vertical diameter (median)	3.3/3.2/4.0
Air bubbles in cysts	Present/absent	24/22
Involvement of thyroid gland	Present/absent	46/0
Airway compression/displacement	Present/absent	35/11
Into retropharyngeal space	Present/absent	31/15
Into mediastinum	Present/absent	8/38

**Table 3:** The analysis of air-containing, the cyst wall, and border of CPSF in newborns of different ages.

**Days**	**Air bubbles in cysts [*****n*** **(%)]**		**Cyst wall [*****n*** **(%)]**		**Border [*****n*** **(%)]**	
	**Presence**	**Absence**	**Thin**	**Thick**	**Well-defined**	**Poorly defined**
1–7	8 (80.0)	2 (20.0)	6 (60.0)	4 (40.0)	9 (90.0)	1 (10.0)
8–14	12 (57.1)	9 (42.9)	9 (42.9)	12 (57.1)	10 (47.6)	11 (52.4)
15–28	4 (26.7)	11 (73.3)	3 (20.0)	12 (80.0)	2 (13.3)	13 (86.7)
*p*	0.008		0.042		0.000	

### Surgical and Pathological Manifestations

All of the patients in this sample underwent surgery to remove the mass and suture the fistula tract, and all pathological reports confirmed the mass being pyriform sinus fistula. The masses are cystic with the entire wall intact and accompanied by cloudy fluid. The histopathologic examination also reported the fistulas were lined with ciliated cell epithelium and squamous cell epithelium with inflammatory cell infiltration. Lastly, giant cells (multinucleated giant cells) reaction was also found. Fibrous tissue hyperplasia was found in the cyst wall of 24 lesions, and thyroid follicles were found in all 46 lesions.

## Discussion

In this study, the US and CT imaging findings of neonatal CPSF were summarized and compared. Our results showed that CPSF commonly presented with a cervical cystic lesion, mainly in the left side, and air bubbles could be recognized in part of the lesions. In comparison with the US examination, air bubbles, the involvement of ipsilateral thyroid, deviation of the airway, and expansion into the mediastinal and retropharyngeal space were more likely to be detected by CT. Moreover, older age was associated with the absence of typical air bubbles, thick cyst wall, and poorly defined border.

To our knowledge, this is the largest study on this issue among newborns to date. The male-to-female ratio was 1.05:1, which indicated the prevalence of neonatal CPSF between males and females was similar (9, 14). All 45 newborns had cervical cystic masses on preoperative imaging examinations, which were consistent with previous studies (7, 8, 11). Neonatal CPSF is actually a cystic dilated fistula on one side, which is related to fetal amniotic fluid intake or neonatal food intake (7). Among the samples, 1 case (1/45) was bilateral, 3 cases (3/45) were right sided, and 41 cases (41/45) were left sided, which indicated that the CPSF mostly develops on the left side of the neck in neonates, which agrees with previous literature (8, 15). The maximum diameter of the cyst was the vertical diameter, which was associated with the anatomical structure of the pyriform sinus fistula (14, 16).

As US does not carry ionizing radiation and it is a simple and convenient process, it should be the first choice for newborns (17). In this study, all lesions were detected by US, showing inhomogeneous cystic low echogenicity and poor internal echogenicity, which were easily confused with other neck infectious lesions and thus led to misdiagnosis. Only 15 cystic masses with air bubbles were diagnosed as CPSF by US clinically. The sign of air-bubbles in the cyst was identified as the characteristic indicator of CPSF in previous studies (18). Our results were comparable with those of the studies, reporting 15 air-bubble cysts diagnosed on US and 24 air-bubble cysts diagnosed on CT. Obviously, CT showed significantly higher detection rates of the air-bubble sign in lesions than US, which may be explained by the fact that the amount of air filled in the lesions was usually small and the individual bias of the US operators. Furthermore, we made the children cry loudly before the CT examination, which could increase the possibility of air entering the focus of CPSF.

CPSF in the neonates showed other distinctive imaging findings on contrast-enhanced CT scan, especially having advantages in terms of confirming the exact location and extent of involvement of a lesion, which is helpful to diagnose the CPSF without specific air bubbles. Based on the anatomical location of the cervical fascial space (19, 20), these lesions were located in the visceral space, as a characteristic imaging manifestation of the disease (18). With the higher spatial and density resolution than the US, CT is more sensitive in detecting the relationship between cysts with the retropharyngeal space, mediastinum, thyroid, and airway. The fistula originates from the pyriform fossa, and descends through the thyroid parenchyma or along with the dorsal thyroid gland, and enters the parathyroid space (14, 19). In these samples, the thyroid glands all presented involved that was consistent with the existence of thyroid follicles in the edge of the lesions after surgical resection. Besides, these lesions induced the compression and displacement of the surrounding structures (21). On contrast-enhanced CT, 35 (35/46) lesions were accompanied by the airway being compressed and shifted. As a result, 10 patients presented with shortness of breath and 31 with pulmonary infection when being sent to the hospital in our cases, which may be attributed to the trachea being compressed and obstructed by the cervical mass and thus respiratory distress. Due to the special location, some lesions that were deep into the retropharyngeal space presented with the shape of “commas.” It is possible that the growth space of the lesions was limited by the surrounding structures such as the airway, carotid artery, and sternocleidomastoid muscle. Consequently, when neonatal CPSF cases are suspected, CT can be used as a further examination to provide more detailed information for the management of CPSF (10).

Interestingly, we found correlations between the age of the patients and some imaging features of the cyst, including the absence of air bubbles in cyst, thick cyst wall, and poorly defined border, in neonatal CPSF. These features meant inflammation and reactive edema from CPSF had occurred in the neonatal period and aggravated with time. However, neonatal CPSF usually involves mild inflammation that does not cause acute suppurative thyroiditis and is characterized by the formation of a large cyst that causes respiratory distress (13). When CPSF continues until childhood, it leads to adhesion to surrounding tissue structures and the opening of the pyriform sinus to the fistula becomes inflammation stenosis progressively (9, 18, 22). That is why the symptoms and management of CPSF differ between neonates and children. All 45 cases in the present study came to the hospital for cervical mass with normal skin color, smooth surface, soft surface, and no tenderness, and most of them presented symptoms in the respiratory system. These findings are in agreement with those of previous reports (6). Therefore, the difficulty of surgical resection of CPSF lesions and the risk of damage to the surrounding tissue increases if early diagnosis and intervention are unachievable (9).

The potential limitations of this study focused on two aspects. First, barium esophagography and MR were not performed in this group of patients. However, barium esophagography was not carried out with the consideration that newborns could not cooperate well with swallowing barium, and there is also a risk of cough complications caused by aspiration (23). Furthermore, MR examination requires a longer period of anesthesia and higher cost. Some researchers reported that MR examinations could not show the presence of minute air bubbles in the cyst sensitively (6). Second, our findings provided preliminary data and adequate justification to further evaluate our recommendations on the diagnosis of CPSF in neonates. Further multi-center studies with a larger sample of neonatal CPSF cases are needed to confirm these findings, and long-term follow-up data are also necessary (24).

In conclusion, US and CT are effective to detect neonatal CPSF, commonly presenting with a cystic lesion in the left of the neck and air bubbles within some lesions. The features of the cyst wall, the border, and the air bubbles inside the cyst on contrast-enhanced CT may be different depending on the age of the patient. CT can provide important supplementary information for the diagnosis of CPSF after the initial US.

## Data Availability Statement

The original contributions presented in the study are included in the article/supplementary materials, further inquiries can be directed to the corresponding authors.

## Ethics Statement

The studies involving human participants were reviewed and approved by the review boards of Hunan Children's Hospital, University of South China. The review board waived the requirement for written informed consent for participation. Written informed consent was not obtained from the minor(s)' legal guardian/next of kin for the publication of any potentially identifiable images or data included in this article.

## Author Contributions

LL, S-PH, and KJ designed this study. LL, D-J-HZ, Y-HX, Q-HM, T-YY, and HL collected and interpreted the patients' data. LL analyzed the data and major contributor to the writing of the article. All authors have read and approved the finalarticle.

## Funding

This study was funded by the Natural Science Foundation of Hunan Province, China (Grant No. 2019JJ40156).

## Conflict of Interest

The authors declare that the research was conducted in the absence of any commercial or financial relationships that could be construed as a potential conflict of interest.

## Publisher's Note

All claims expressed in this article are solely those of the authors and do not necessarily represent those of their affiliated organizations, or those of the publisher, the editors and the reviewers. Any product that may be evaluated in this article, or claim that may be made by its manufacturer, is not guaranteed or endorsed by the publisher.

## References

[1] Goins MR Beasley MS. Pediatric neck masses. Oral Maxillofac Surg Clin North Am. (2012) 24:457–68. 10.1016/j.coms.2012.05.006 22857718

[2] Goff CJ Allred C Glade RS. Current management of congenital branchial cleft cysts, sinuses, and fistulae. Curr Opin Otolaryngol Head Neck Surg. (2012) 20:533–9. 10.1097/MOO.0b013e32835873fb 23128685

[3] Nicoucar K Giger R Jaecklin T Pope HG Jr. Dulguerov P. Management of congenital third branchial arch anomalies: a systematic review. Otolaryngol Head Neck Surg. (2010) 142:21–8 e2. 10.1016/j.otohns.2009.09.001 20096218

[4] de Buys Roessingh AS Quintal MC Dubois J Bensoussan AL. Obstructive neonatal respiratory distress: infected pyriform sinus cyst. J Pediatr Surg. (2008) 43:E5–8. 10.1016/j.jpedsurg.2007.12.071 18485936

[5] Miyauchi A Matsuzuka F Takai S Kuma K Kosaki G. Piriform sinus fistula. A route of infection in acute suppurative thyroiditis. Arch Surg. (1981) 116:66–9. 10.1001/archsurg.1981.01380130044010 7469735

[6] Pan J Zou Y Li L Yang TY Yang JL Hu C. Clinical and imaging differences between neonates and children with pyriform sinus fistula: which is preferred for diagnosis, computed tomography, or barium esophagography? J Pediatr Surg. (2017) 52:1878–81. 10.1016/j.jpedsurg.2017.08.006 28886900

[7] China M. Child health association minimally invasive chapter pediatric ORLG. [Clinical practice guidelines for the diagnosis and management of congenital pyriform sinus fistula in children]. Lin Chung Er Bi Yan Hou Tou Jing Wai Ke Za Zhi. (2020) 34:1060–4. 10.13201/j.issn.2096-7993 33254335 PMC10127779

[8] Liu Z Han J Fu F Liu HS He QM Zhong W. How to make an accurate diagnosis of fetal pyriform sinus fistula in utero: experience at a single medical center in mainland China. Eur J Obstet Gynecol Reprod Biol. (2018) 228:76–81. 10.1016/j.ejogrb.2018.05.039 29909267

[9] Zhu H Xiao X Zheng S Shen C. Diagnosis and management of pyriform sinus cyst in neonates: 16-year experience at a single center. J Pediatr Surg. (2017) 52:1989–93. 10.1016/j.jpedsurg.2017.08.041 28987714

[10] Xia L Lin Z Lin X Wang Y Zhu L Lin J. The treatment of congenital pyriform sinus fistula: a single-center experience. Pediatr Surg Int. (2020) 36:779–88. 10.1007/s00383-020-04676-2 32424498

[11] Leboulanger N Ruellan K Nevoux J Pezzettigotta S Denoyelle F Roger G. Neonatal vs delayed-onset fourth branchial pouch anomalies: therapeutic implications. Arch Otolaryngol Head Neck Surg. (2010) 136:885–90. 10.1001/archoto.2010.148 20855681

[12] Teng Y Huang S Chen G Xian Z Han S Li L. Congenital pyriform sinus fistula presenting as a neck abscess in a newborn: a case report. Medicine (Baltimore). (2019) 98:e17784. 10.1097/MD.0000000000017784 31689849 PMC6946197

[13] Amano H Uchida H Sato K Kawashima H Tanaka Y Takazawa S. Differences in the characteristics and management of pyriform sinus fistula between neonates and young children. Pediatr Surg Int. (2012) 28:15–20. 10.1007/s00383-011-3008-z 22009210

[14] Rea PA Hartley BE Bailey CM. Third and fourth branchial pouch anomalies. J Laryngol Otol. (2004) 118:19–24. 10.1258/002221504322731574 14979967

[15] Li W Xu H Zhao L Li X. Branchial anomalies in children: a report of 105 surgical cases. Int J Pediatr Otorhinolaryngol. (2018) 104:14–8. 10.1016/j.ijporl.2017.10.035 29287855

[16] Gong XX Chen LS Xu MM Huang SL Zhang B Liang L. [Clinical anatomic study on the segment and adjacent of tract of congenital pyriform sinus fistula]. Zhonghua Er Bi Yan Hou Tou Jing Wai Ke Za Zhi. (2018) 53:604–9. 10.3760/cma.j.issn.1673-0860.2018.08.009 30121999

[17] Liu Z Tang SS. Diagnosis of pyriform sinus fistula in children via ultrasonography. Am J Otolaryngol. (2013) 34:579–81. 10.1016/j.amjoto.2013.01.010 23790565

[18] Han Z Tai J Gao J Wang S Yu T Peng Y. MRI in children with pyriform sinus fistula. J Magn Reson Imaging. (2021) 53:85–95. 10.1002/jmri.27325 32896061

[19] Warshafsky D Goldenberg D Kanekar SG. Imaging anatomy of deep neck spaces. Otolaryngol Clin North Am. (2012) 45:1203–21. 10.1016/j.otc.2012.08.001 23153745

[20] Coley BD. Caffey's pediatric diagnostic imaging. Thirteenth edition. Philadelphia, PA: Elsevier (2019). p. 119–21

[21] Sheng Q Lv Z Xu W Liu J. Differences in the diagnosis and management of pyriform sinus fistula between newborns and children. Sci Rep. (2019) 9:18497. 10.1038/s41598-019-55050-9 31811210 PMC6898025

[22] Chen T Ge G Chen J Zhao X Sheng Q Zhu L. Pyriform sinus fistula in children: preferred imaging modality and risk factors for diagnostic delay. Front Pediatr. (2020) 8:575812. 10.3389/fped.2020.575812 33194907 PMC7661853

[23] Hosokawa T Yamada Y Takahashi H Tanami Y Sato Y Hosokawa M. Optimal timing of the first barium swallow examination for diagnosis of pyriform sinus fistula. AJR Am J Roentgenol. (2018) 211:1122–7. 10.2214/AJR.18.19841 30240303

[24] Zhang P Tian X. Recurrent neck lesions secondary to pyriform sinus fistula. Eur Arch Otorhinolaryngol. (2016) 273:735–9. 10.1007/s00405-015-3572-2 25708412

